# Tonotopic Selectivity in Cats and Humans: Electrophysiology and Psychophysics

**DOI:** 10.1007/s10162-022-00851-5

**Published:** 2022-06-13

**Authors:** Francois Guérit, John C. Middlebrooks, Matthew L. Richardson, Akshat Arneja, Andrew J. Harland, Robin Gransier, Jan Wouters, Robert P. Carlyon

**Affiliations:** 1 Cambridge Hearing Group, MRC Cognition & Brain Sciences Unit, University of Cambridge, Cambridge, England; 2 Department of Otolaryngology, University of California at Irvine, Irvine, CA USA; 3 Department of Neurobiology and Behavior, University of California at Irvine, Irvine, CA USA; 4 Department of Cognitive Sciences, University of California at Irvine, Irvine, CA USA; 5 Department of Biomedical Engineering, University of California at Irvine, Irvine, CA USA; 6 Dept. of Neurosciences, ExpORL, Leuven, Louvain, KU Belgium

**Keywords:** cortical onset response, tonotopic selectivity, cat, human, psychophysics, electrophysiology

## Abstract

We describe a scalp-recorded measure of tonotopic selectivity, the “cortical onset response” (COR) and compare the results between humans and cats. The COR results, in turn, were compared with psychophysical masked-detection thresholds obtained using similar stimuli and obtained from both species. The COR consisted of averaged responses elicited by 50-ms tone-burst probes presented at 1-s intervals against a continuous noise masker. The noise masker had a bandwidth of 1 or 1/8th octave, geometrically centred on 4000 Hz for humans and on 8000 Hz for cats. The probe frequency was either − 0.5, − 0.25, 0, 0.25 or 0.5 octaves re the masker centre frequency. The COR was larger for probe frequencies more distant from the centre frequency of the masker, and this effect was greater for the 1/8th-octave than for the 1-octave masker. This pattern broadly reflected the masked excitation patterns obtained psychophysically with similar stimuli in both species. However, the positive signal-to-noise ratio used to obtain reliable COR measures meant that some aspects of the data differed from those obtained psychophysically, in a way that could be partly explained by the upward spread of the probe’s excitation pattern. Our psychophysical measurements also showed that the auditory filter width obtained at 8000 Hz using notched-noise maskers was slightly wider in cat than previous measures from humans. We argue that although conclusions from COR measures differ in some ways from conclusions based on psychophysics, the COR measures provide an objective, noninvasive, valid measure of tonotopic selectivity that does not require training and that may be applied to acoustic and cochlear-implant experiments in humans and laboratory animals.

## Introduction

Tonotopic organisation is a fundamental principle of the auditory system. In normal-hearing listeners, high- and low-frequency sounds activate more-or-less discrete populations of auditory nerve fibres that innervate respectively basal and apical portions of the cochlea (Liberman 1982; Narayan et al. 1998). The resulting frequency-based neural organisation is conveyed through successive levels of the auditory pathway from cochlear nucleus to auditory cortex. This tonotopic selectivity is reduced in cases of sensory hearing loss, due to the loss of the cochlear outer hair cells (e.g. Dallos and Harris 1978). It is even more severely degraded when normal cochlear mechanics and synaptic transmission are replaced by electrical stimulation with a cochlear implant (CI). That degradation is due to the considerable spread of current along the cochlea and of the resulting spread of excitation across the auditory nerve array (e.g. cat: Middlebrooks and Snyder 2007; George et al. 2014; human: Nelson et al. 2008; Bierer and Faulkner 2010; Cosentino et al. 2015).

We aim to develop a noninvasive electrophysiological measure of tonotopic spread of activation that can be applied in synergistic animal and human experiments. The measure ideally could be applied not only to acoustic stimulation but also to prosthetic hearing replacements including CIs and novel methods of stimulating the auditory nerve that are in development (e.g. intraneural electrodes: Middlebrooks and Snyder 2007, 2010; optogenetic stimulation: Dieter et al. 2019). Careful design of such an objective measure could also aid the evaluation of other (e.g. pharmaceutical) interventions using animals and could provide an important link to human perception. We believe that, to do so effectively, the objective measure should be relatable to parallel perceptual measures that use similar stimuli and paradigms, both in human and nonhuman species. For example, tonotopic selectivity of CIs is usually measured in newly-deafened, anaesthetised, cats or guinea pigs using single-pulse stimuli (Synder et al. 1990, 2004), whereas human studies, typically in long-term deaf subjects, usually employ longer-duration pulse trains and psychophysical tasks. These differences might account for apparently discrepant findings such as the success of so-called focused methods of stimulation in improving tonotopic selectivity in cats and guinea pigs (Raggio and Schreiner 1999; Rebscher et al. 2001; Bierer and Middlebrooks 2002; Middlebrooks and Bierer 2002; Snyder et al. 2004, 2008; Bonham and Litvak 2008; Bierer et al. 2010; George et al. 2014), compared to the modest and variable gains observed in humans (Boëx et al. 2003; Kwon and van den Honert 2006; Chatterjee et al. 2006; Bierer and Faulkner 2010; Srinivasan et al. 2010; Chua et al. 2011; Fielden et al. 2013; Marozeau et al. 2015; Padilla and Landsberger 2016; Carlyon et al. 2017; Luo et al. 2020).

The present report describes results obtained with an objective estimate of tonotopic selectivity obtained with scalp-measured responses. This “cortical onset response (COR)” was obtained in both cats and humans and was compared with parallel psychophysical measurements in both species. Both the objective and perceptual measures were obtained using acoustic tones in maskers consisting of narrow (1/8-oct) and wider (1-oct) bands of noise. In all cases, the differences in the tonotopic spread of activation between the two masker bandwidths were prominent, supporting the use of the COR as an objective measure of spread of excitation, both in normal acoustic hearing and in CIs. The results showed substantial quantitative agreement between feline and human subjects and between COR and psychophysical measures. An exception to this occurred for tone frequencies on the edge of the 1-octave masker frequency band, where the COR revealed less masking than did the psychophysical measures. A nonlinear model of peripheral auditory filtering suggested this was due to the use of supra-threshold tones in the COR recordings and to the upper edge of the tones’ excitation patterns contributing to the COR but not to detection. Psychophysical measurements of the auditory filter width using notched-noise stimuli showed that the cat auditory filter was approximately 22 % wider than that of humans, thereby contributing to the ongoing debate on differences in tonotopic selectivity between humans and animals (e.g. Shera et al. 2010; Sumner et al. 2018).

We conclude that the COR provides a noninvasive objective measure of tonotopic selectivity that is suitable for acoustic, electric and other forms of auditory stimulation, can be measured in laboratory animals and humans, and is consistent with psychophysical measures obtained with similar stimuli and the same species. Unlike psychophysical measures, it does not require training, thereby providing a time-efficient method that can be applied to a wide range of species.

## Electrophysiological Measures 

### General Methods

#### Narrow-Band Versus Wide-Band Masker

**Fig. 1:**
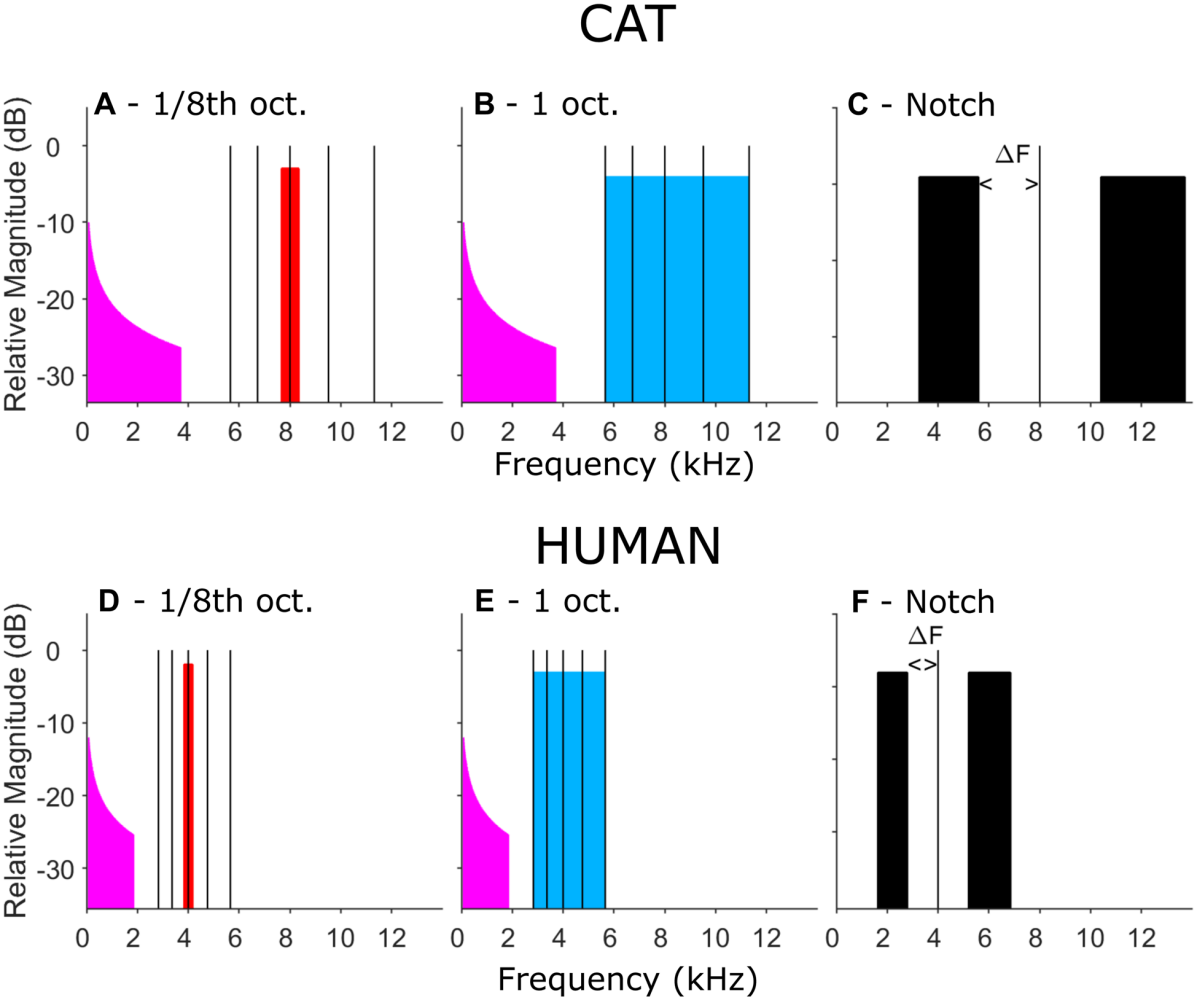
Stimulus spectra. **A**, **B** 1/8th-oct (**A**, red) and 1-oct (**B**, blue) maskers for cats, centred on 8 kHz. Pink noise (magenta) was used to mask quadratic distortion products. Vertical black lines indicate probe frequencies, which were presented one at a time at varying levels. **C** Notched-noise stimuli for cats. Lower- and upper-frequency masker bands were held constant in width equal to the lower and upper halves of the 1-oct masker. The probe frequency was fixed at 8 kHz and the notch width varied among 20, 40 and 60 % (ΔF = 10, 20, and 30 %) of the centre frequency. **D**, **E** 1/8-oct, 1-oct, and notched maskers for humans, all centred on 4 kHz. F. Notched-noise stimuli used for humans

**Fig. 2:**
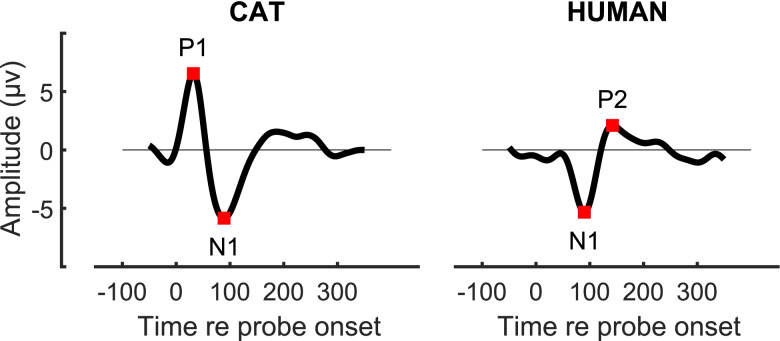
Examples of cat (left panel) and human (right panel) cortical onset response (COR) waveforms

Our stimulus design is illustrated schematically in Fig. 1. It compares the selectivity of a narrow-band (1/8th-octave, Fig. 1A and D, shown in red) with that of a wider-band (1-octave) masker (Fig. 1B and E, shown in blue), using 50-ms pure-tone probes at various frequencies (black lines). The maskers were presented continuously at a fixed level and frequency, and the probes were presented at 1-s intervals. We measured the COR to these probes in both cats and humans. Typical COR waveforms for each species are shown in Fig. 2. The COR can be elicited either in response to the onset/offset of a sound or to a change in an ongoing sound (cf. review by Näätänen and Picton 1987). We measured the COR to tones presented during the noise maskers (simultaneous masking) rather than in forward masking to avoid responses to the masker offset (Brattico et al. 2003) and to minimise confusion effects between the narrowband masker and probe that have been observed in psychophysical forward masking experiments (e.g. Moore et al. 1984; Neff 1985; McKay 2012; Cosentino et al. 2015). Simultaneous masking has often been used for psychophysical measurements of the width of the auditory filter in several species (e.g. human: Patterson et al. 1982; Glasberg and Moore 1990; cat: Pickles 1975; ferret: Alves-Pinto et al. 2016; Sumner et al. 2018).

In the cat experiment, the maskers and the set of probe frequencies were centred on 8 kHz (top row, Fig. 1). That frequency band was chosen to activate approximately the tonotopic region that is stimulated by a cochlear implant that can be positioned in the cat’s basal cochlear turn. The maskers were created in the frequency domain with flat amplitude spectra and random phase spectra between brick-wall cut-offs of 7.66 to 8.35 kHz for the 1/8th-oct masker or 5.66 to 11.31 kHz for the 1-oct masker. The probe-tone frequencies were 5.66, 6.73, 8.00, 9.51, and 11.31 kHz, which is in the range of ½ oct below to ½ oct above 8 kHz in ¼-oct steps. Pink noise (having a spectrum with equal energy per octave) was added to the masker to mask quadratic combination tones arising from the interaction of the probes with the masker. The pink noise was bandpass filtered between 85 and 3700 Hz and had a level per 1/6th octave that was 25.3 dB below the overall level of the 1/8-octave masker. Because the width of the auditory filter is roughly proportional to centre frequency for frequencies above 500 Hz, and—at least in humans—is roughly 1/6th-octave wide, this means that the pink noise would mask distortion products for a wide range of frequencies in its passband and where the level of the distortion product is more than about 25 dB lower than that of the 1/8th-octave noise. The actual level of quadratic distortion products depends on the levels and frequencies of the primaries, but for the levels and frequencies used here are likely to be at least 40 dB below that of the primaries (Goldstein 1967; Hall 1972). Masking of cubic distortion products by the pink noise is discussed in detail in the “25” section.

At the beginning of each session, the COR was recorded in response to nonmasked 8.0 kHz probe tones at varying levels. In most cases, a level of 75 dB SPL was at or near the top of the steep part of the COR-versus-level function and was used as the probe level for subsequent measures; in one cat, the dynamic range encompassed slightly higher levels, and a probe level of 80 dB SPL was used, with correspondingly increased masker levels. The probe levels were chosen to fall in a portion of the level-growth function such that there would be a large COR in the absence of the masker and so that a masker-induced reduction in the effective probe level would result in a reduction of the COR amplitude. The 1/8th-oct masker level was held at 65 dB SPL, which was a + 10-dB probe/masker ratio. At that masker level, the COR elicited by the 8-kHz probe typically was about 40–50 % of that in the no-masker condition. In pilot experiments, the 1-oct masker level was set at 73.2 dB SPL, which was estimated to equalise the energy between 1/8th- and 1-oct maskers in a human-sized auditory filter centred on 8 kHz (estimated to be 888 Hz; Glasberg and Moore 1990). That masker level, however, tended to reduce the response to an 8-kHz tone to near the noise floor. Therefore, in subsequent experiments, we held the 1-oct masker at 70 dB SPL; an even lower level, 68 dB SPL, was used for two cats.

In the human experiments, the stimulus spectra were shifted down an octave (i.e., halved in frequency) relative to the cat stimuli to accommodate the difference in audible range of the two species (Fig. 1, bottom row). Maskers were centred on 4 kHz, passbands were 3.83 to 4.18 kHz (1/8 oct) and 2.83 to 5.66 kHz (1 oct), probe frequencies were 2.83, 3.36, 4.00, 4.76 and 5.66 kHz, and the upper cutoff of the pink-noise masker was 1.86 kHz. The CORs were measured in response to 70-dB SPL, 50-ms probes. For the 1/8th-oct masker, CORs were obtained at four masker levels: 48, 52, 56, and 60 dB SPL. The 1-oct masker levels were 7.9 dB higher, so that the 1/8th- and 1-oct. maskers would have the same energy passing through an auditory filter centred on 4 kHz in NH listeners (456 Hz; Glasberg and Moore 1990). However, at all except the highest masker level, there were instances where no masking (reduction in COR amplitude) was observed, and so we describe only the results obtained for the 60 dB/ERB masker (i.e. 60 dB SPL and 67.9 dB SPL for the 1/8th- and 1-oct. maskers, respectively). At this level, the signal-to-masker ratio was 10 dB, identical to that used for the cat. The full dataset is available in an online repository (Zenodo, http://doi.org/10.5281/zenodo.5543925).

#### Notched-Noise Masker

In human listeners, in addition to the comparison between 1/8th-oct and 1-oct maskers, we measured tonotopic selectivity of the COR using notched-noise maskers (Patterson 1976), which, although less transferrable to CI stimulation, minimises effects of off-frequency listening and of cochlear distortion products. This provided an electrophysiological measure of the auditory filter width that could be compared with published psychophysical measures and with new psychophysical measures in the present study. The probe tone was fixed at 4 kHz and 70 dB SPL, and the continuous masker consisted of the previously used 60 dB SPL/ERB, 1-oct noise band centred logarithmically on 4 kHz. The noise band was then split into two halves at 4 kHz, and the upper and lower portions were shifted successively further apart, creating notches of varying widths. The widths of the upper and lower halves of the noise band (in Hz) were held constant. The notch widths were quantified by Δf, which was the frequency difference from 4 kHz to the nearest edge of the noise band above or below 4 kHz. The tested Δf values were 0, 10, 20 and 30 % of the centre frequency; the 0 % condition was identical to the 1-oct noise band masker. The spectra for Δf = 30 % are illustrated in Fig. 1C (for cats, only used for psychophysical experiments described in a later section) and Fig. 1F (for humans, used for both CORs and psychophysics).

For both species and for both COR and psychophysical measurements, the data were analysed using IBM SPSS Statistics v. 26 for analyses of variance and the R software package for other analyses. Note that the SPSS package does not distinguish between *p* values lower than 0.001 and so such values are reported as “*p* < 0.001”.

### Methods—COR Recordings in Cats

#### Animals

Domestic short-haired cats (*Felis catus*) were obtained from a breeding colony at the University of California at Davis. No hearing deficits were evident. All procedures were in accordance with the NIH Animal Welfare Guidelines and with protocols approved by the Institutional Animal Care and Use Committee at the University of California at Irvine.

The COR recordings were made from 7 adult cats. Three of these were neutered males that had been trained in the detection of tones in noise and provided the psychophysical data described later in this paper. They were 21–24 months of age and weighed 4.4–6.0 kg at the time of COR recordings. The remaining 4 cats were untrained females ranging from 9 to 22 months of age and weighing 2.8–4.3 kg at the time of COR recordings.

Cats were sedated for the COR recording. A light level of anaesthesia was induced with an intramuscular injection of ketamine (20 mg/kg) and acepromazine (1 mg/kg). At those doses, eye-blink or limb-withdrawal reflexes sometimes could be elicited, but there were no spontaneous movements. One or more supplemental doses of ketamine alone were given during the experiment if needed to maintain an immobile state. Recording sessions lasted 1 to 2 h.

#### Setup

The recordings were conducted in a single-wall sound-attenuating chamber. Stimulus generation, waveform recording, and experimental control used System III hardware from Tucker-Davis Technologies (TDT; Alachua, FL) controlled by custom MATLAB scripts (The Mathworks, Natick, MA) on a Windows-based personal computer. Sounds were generated with 24-bit precision at a sample rate of 97.7 kHz. The 1/8th-oct and 1-oct maskers and the probe tones were presented through a Radio Shack horn tweeter, and the pink noise was presented through a 3-inch co-axial speaker (Pioneer TS-A878), both speakers located 20 cm to the left of the cat’s left ear. The speakers were calibrated in the sound field prior to each testing session using a ½-inch precision microphone (ACO Pacific) in the absence of the cat. Acoustic probes for calibration consisted of Golay codes (Zhou et al. 1992) for masker and pink-noise spectra and tones for the tonal stimuli. Correction spectra were derived for the speakers, used to equalise sound levels at known levels re 20 µPa across the stimulus bands. Scalp-recorded waveforms were obtained with hypodermic needle electrodes placed over the right hemisphere (active), behind the left ear (reference), and on the back (ground). Waveforms were amplified with a TDT low-impedance headstage, digitised at a rate of 24.4 kHz, displayed, and stored to disk.

Each block of measurements began with the onset of a continuous masker (or a period of no masker). After 1 s, the sequence of 50-ms tone pips (the probes) was begun at a rate of 1/s. The tone pips had 5-ms cosine-squared onset and offset ramps. Each of the 5 probe frequencies was presented once in a random order, and then again in another random order, and so on for 100 repetitions of each frequency. The masker condition was held constant for each block of 500 tone presentations.

Offline analysis employed custom MATLAB scripts. Waveforms were filtered between 3 and 20 Hz. Individual waveforms that exceeded 4 times the root-mean-squared background level within the 0-to-120 ms analysis window were excluded from further analysis; this was on average only 0.6 % of waveforms. Then, the remaining ~ 100 waveforms for each masker and tone condition were averaged. The response to the onset of a tone consisted of a positive peak (P1), followed by a negative peak (N1) (Fig. 2). A second positive peak (P2) often was present, but it was not as consistent in latency and morphology as were the P1 and N1. The P1 is regarded as a middle-latency component and has been attributed to activity in the primary auditory cortex (Kaga et al. 1980) or the thalamocortical projection (McGee et al. 1991, 1992). Latencies of peaks were given by the time of reversal of the slope of the waveform within the specified criterion window. Based on inspection of waveforms in multiple cats and conditions, we set latency criteria of 15 to 55 ms for P1 and 55 to 120 ms for N1. We quantified the magnitudes of the COR by computing the amplitudes of P1 minus N1 (in µV) for each stimulus condition.

### Methods—COR Recordings in Humans

#### Participants

There were two groups of human participants. Group A was tested on the 1/8th-oct and 1-oct COR measurements. It originally consisted of 13 participants but 3 were rejected for having CORs under 3 µV at all frequencies, and so all plots and statistical analyses from this group are based on the remaining 10 participants. In another group, group B (*N* = 12), we collected data for the notched-noise COR measurements, as well as data for the COR to the probe alone at various levels and frequencies. Group B also participated in the psychophysical experiments reported later in this manuscript. Participants were paid for their participation except for author AJH, who was the only participant common to both groups. Experimental procedures were approved by the Cambridge Psychology Research Ethics Committee (project 2017.085), and written informed consent was collected prior to any testing. An audiogram revealed that all participants had thresholds of 20 dB HL or lower at all audiometric frequencies. Stimulation was always monaural, with the numbers of right and left ears balanced across participants (7 right and 6 left in group A, 6 right and 6 left in group B).

#### Setup

All stimuli were generated using custom MATLAB scripts, played through a RME Fireface UCX sound card, TDT HB7 headphone drivers, and an ER2 insert phone. Stimuli were calibrated using an ear coupler (GRASS) and a Hewlett Packard HP3561A dynamic signal analyser. Potentials were recorded from the scalp with an 8-channel Biosemi system with electrodes located at P7, P8, Oz, Cz, AF3, AF4 and above/below the left eye. Four electrodes (P7, P8, P10, Cz) covered respectively the left/right mastoids, the back of the head and the vertex, which are typical electrode positions for COR recordings. The other four electrodes were closer to the eyes (AF3/AF4 are in the frontal area), so as to detect eye artefacts. Scalp potentials were recorded at a sampling rate of 2048 Hz, and the cutoff of the anti-aliasing low-pass filter was 1/5th of this rate (~ 400 Hz). Digital triggers were routed from the sound card to the triggering input of the Biosemi system via a custom-built triggering interface (Mao et al. 2018, https://github.com/d-mao/trigger-box). The various conditions were marked by distinct digital trigger codes.

Participants were seated in a reclined armchair, inside a double-walled, electrically shielded, sound-attenuating booth. They watched TV shows with subtitles while undergoing the test.

For each of the conditions, a sweep of 13 fixed-frequency probes with 1-s stimulus onset asynchronies (SOAs) was created. Probes were 50-ms sinusoids with 2-ms onset and offset ramps. For the masked conditions, the masker started simultaneously with the first probe in each sweep and lasted for 13 s; the first probe in each sweep was ignored in the analysis because its onset coincided with the start of the masker. There was a silent gap of approximately 1.2 s between each sweep (1 s plus computing time). All of the sweeps so obtained (all the various probe frequencies/levels and maskers) were randomly interleaved in one block. Each block was repeated 20 times, with the order of the sweeps within each block randomised afresh.

#### Analysis

The data were analysed using the Fieldtrip toolbox (Oostenveld et al. 2011) and custom MATLAB scripts. First, the difference between the contralateral mastoid electrode (P7 if stimulation was in the right ear, P8 for left-ear stimulation) and the vertex electrode (Cz) was computed. We picked this montage over other possible options (ipsilateral, back of the head, etc.) because it led to strong CORs in pilot measurements and because that montage is often used for cochlear implant recordings (due to its maximal distance from the implant itself, e.g. Brown et al. 2008). For each condition, 240 epochs were created by windowing from 200 ms before to 600 ms after each probe onset (12 probes per block and 20 block repeats). All epochs were then filtered between 1 and 20 Hz with a finite-impulse-response filter with a 1-Hz transition bandwidth and 2-s padding. For artefact rejection, the difference between the channel below and above the eye was computed. For each participant, a z-score was computed from the amplitude variation across the whole duration of their recording. Epochs that contained extreme artefacts (z-score > 4) were rejected, corresponding to between 5 and 15 % of the total number of epochs, depending on the condition. The remaining artefact-free epochs were averaged using the 200 ms before the probe onset as baseline. The amplitudes of the N1 and P2 peaks of the COR were obtained by identifying a minimum and maximum in the [50–150 ms] and [100–200 ms] windows after the probe onset. The response amplitude was given by P2 minus N1.

### Results

#### Level-Growth Functions

Level-growth functions can be useful when comparing COR measures, where the dependent variable is the cortical response, to psychophysical measures where the dependent variable is the threshold probe level. Specifically, they allow one to calculate the reduction in probe level in quiet that produces the same reduction in COR as produced by a given masker. They are also useful for verifying that the COR is in a range of the input–output function where an effective reduction in the probe level, produced by the masker, would be reflected in a substantial reduction in the COR.

**Fig. 3:**
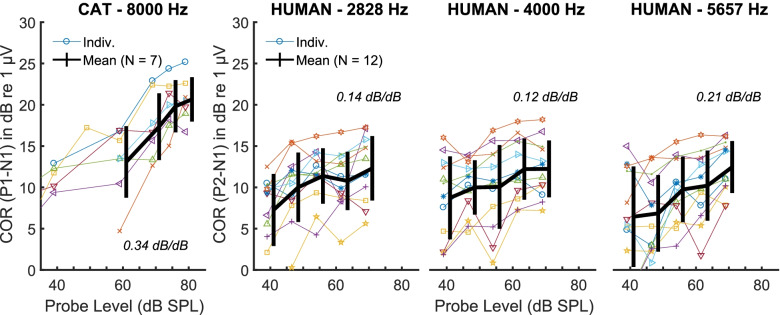
Growth of the unmasked COR with probe level in cats (left panel) and humans (right three panels). For the humans, we measured the COR level-growth at 2828, 4000 and 5657 Hz. Human data is from participant group B. Individual symbols/colours match those of Fig. 5 (notched-noise results)

Level-growth functions from the cat using the 8-kHz probe are shown in the left-hand plot in Fig. 3. The overall size of the COR differs among animals (coloured lines) but the slopes are broadly similar, with an average value of 0.34 on dB/dB co-ordinates in the top 35 dB of input levels used, i.e. between 40 and 75 dB SPL. The plot confirms that our choice of probe levels of 75 dB and of (in one instance) 80 dB fulfilled our criteria of producing substantial CORs and of lying on a steep part of the growth function.

Level-growth functions for humans for probe frequencies of 2828, 4000 and 5657 Hz are shown in the three right-hand plots in Fig. 3. The overall size of the COR varied across participants, but the slopes of the growth functions were broadly similar across participants although shallower than for the cat. A linear mixed model analysis (lmerTest, Kuznetsova et al. 2017) on the COR amplitudes revealed a significant effect of level (*F*(1, 156) = 107.9, *p* < 0.001), but no effect of frequency (*F*(1, 12) = 2.997, *p* = 0.109) nor any interaction (*F*(1, 156) = 2.679, *p* = 0.104). The final model relating the COR in dB re 1 µV to probe level in dB SPL and frequency for each participant *p* was *COR* = *x.level* + *y*_*p*_*.log10(freq),* and the slope *x* was 0.16 dB/dB with 95 % confidence intervals between 0.13 and 0.19 dB/dB.

#### Bandpass Maskers

**Fig. 4:**
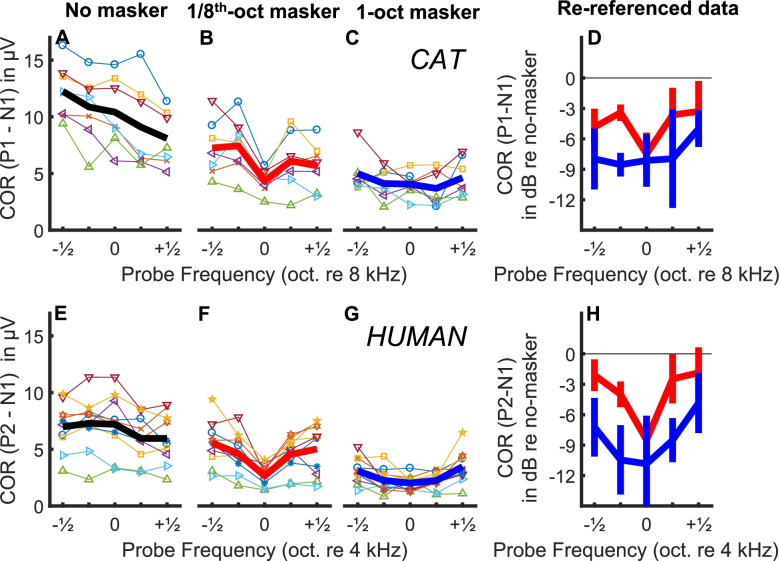
Masked COR patterns in humans (group A) and cats. **A**–**C** Individual (thin lines) and group (thick line) cat COR masked patterns, in µV. **D** Mean masked COR when re-referenced to the no-masker condition. Blue line is for the 1-oct masker, red line is for the 1/8th-oct masker. Error bars indicate ± 1 SD. **E**–**H** Same as **A**–**D** but in humans

The dependence of COR magnitudes on probe frequency was clearly distinguishable between the two maskers both for cats (Fig. 4A–C) and humans (Fig. 4E–G). In each panel, thin coloured lines represent individuals, and the thick line represents the grand mean for each stimulus condition. As was true of the level plots, there was considerable variation in overall COR magnitudes across individuals. Nevertheless, for both species, the 1/8th-oct masker produced a marked reduction of the response to the centre probe-frequency with a steep recovery of responses to off-centre frequencies (Fig. 4B, F). The 1-oct masker produced rather uniform reduction of responses across all the probe frequencies (Fig. 4C, G).

The grand means (± 1 s.d.) of COR magnitudes were expressed in dB relative to the no-masker condition and are plotted in Fig. 4D, H. In the cat, the grand means differed between the two masker conditions by only ~ 0.6 dB for the 8-kHz probe. That indicates that the 1/8-oct band at 65 dB SPL produced about the same amount of masking at 8 kHz as did the 1-oct noise band, which had a level of 70 dB SPL for five cats and 68 dB SPL for two cats. Note, however, that these levels of the 1-oct band are 3.2 and 5.2 dB lower than that needed to produce the same level as the 1/8-oct band at the output of a human auditory filter centred on 8 kHz (Glasberg and Moore 1990). Possible reasons for this difference are described in the “25” section.

In the 1/8-oct masker condition, cat responses to the off-centre-frequency probes showed a sharp release from masking, increasing by ~ 4 dB in magnitude for probes at ¼-oct and ½-oct on either side of 8 kHz. In the 1-oct masker condition, in contrast, responses were within a range of ~ 0.6 dB for all probe frequencies within most of the masker bandwidth; masking on the upper edge of the 1-oct masker, at 11.3 kHz probe was considerably less than for other probe frequencies, similar to the finding in the human experiments (Fig. 4H). Analysis of variance (ANOVA) of the cat magnitudes in dB (re unmasked COR) showed significant main effects of masker bandwidth (*F*(1,6) = 14.9, *p* = 0.0084) and probe frequency (*F*(4,24) = 3.8, *p* = 0.016) and a significant two-way interaction (*F*(4,24) = 3.6, *p* = 0.019), reflecting the greater dependence of COR amplitude on probe frequency for the 1/8th-oct. masker than for the 1-oct. masker. Bonferroni-corrected post hoc comparisons showed significant differences in COR magnitudes between the two masker conditions for probe frequencies of 6.7 and 9.5 kHz (*p* < 0.01) but not for 5.7, 8.0 or 11.3 kHz (*p* > 0.05).

Similar to the cat results, statistical analysis of the human results showed significant main effects of masker type (*F*(1, 9) = 49.0, *p* < 0.001) and probe frequency (*F*(4, 36) = 19.3, *p* < 0.001) and a significant interaction between masker type and probe frequency (*F*(4, 36) = 3.9, *p* = 0.011). This reflects that the COR measure is sensitive enough to distinguish between the two masker widths and to reveal the greater dependence of COR on probe frequency for the 1/8th oct. masker than for the 1-oct. masker. Bonferroni-corrected post hoc comparisons showed significant differences in COR magnitudes between the two masker conditions for probe frequencies of 2.8, 3.4 and 4.8 kHz (*p* < 0.01) but not for 4.0, or 5.7 kHz (*p* > 0.05). The COR did vary somewhat with probe frequency even for the 1-oct masker, as revealed by a significant effect of probe frequency when the 1-oct data alone were analysed using a one-way ANOVA (F(4,36) = 8.2, *p* < 0.001). Paired comparisons revealed that the only differences that remained significant after Bonferroni correction involved the highest probe frequency (5657 Hz), for which the COR was significantly larger than for all other probe frequencies except the lowest (2828 Hz). In the “25” section we attribute this, and the similar finding obtained for cats, to the upward spread of excitation produced by a supra-threshold probe presented on the upper edge of the 1-oct masker.

#### Notched-Noise Masker

**Fig. 5:**
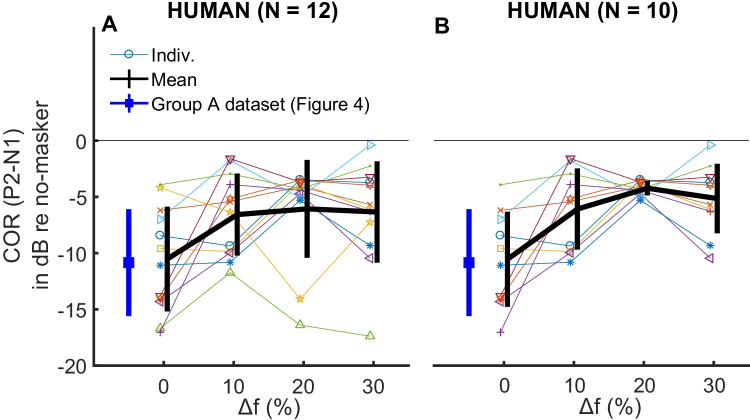
Masked COR patterns in humans (group B) for the different notched-noise conditions. Results are shown in dB relative to the unmasked COR. Left panel shows the results for all participants in group B, with the black thick line showing the mean ± 1 standard deviation. For comparison, we show the masked COR obtained in group A with the 1-oct masker and a 4-kHz probe frequency in blue. That condition and the notched-noise condition with 0 % Δf are identical in terms of stimulation. Right panel shows the same results when removing two outlier participants with high amounts of masking for Δf of 20 and 30 %. Individual colours and symbols match those of Fig. 3

The results of the notched-noise measurements obtained with all members of group B of the human participants are shown in Fig. 5A. For comparison, the blue unconnected symbol to the left of Fig. 5 shows the results obtained in group A with the 1-oct masker at 60 dB SPL/ERB and a 4-kHz probe, for which the stimuli were identical to the 0 % notch-width condition. The average reduction in amplitude relative to the no-mask condition is similar to that in this new dataset (*t*(20) = 0.16, *p* = 0.88), and is about 10–11 dB. There was an overall effect of changing the notch width on the COR amplitude (*F*(3, 33) = 5.0, *p* = 0.0056). Nevertheless, the response to the tone was very similar for values of Δf between 10 and 30 %, and an ANOVA based on those data alone revealed no significant effect of notch width (*F*(2,22) = 0.113, *p* = 0.871) and no post hoc comparisons between conditions were significant. This result differs from the psychophysical findings in which thresholds continue to drop up to the widest notch width (e.g. Patterson et al. 1982; Glasberg and Moore 1990). Two outlier participants showed considerable masking at Δf of 20 % and 30 %; removing them from the analysis (Fig. 5B) did not change the statistical results (Fig. 5B; *F*(3, 27) = 10.5, *p* < 0.001, all notch widths included) but, numerically, made the COR amplitude continue to increase up to Δf = 20 %.

## Psychophysical Measures

### Overview and Stimulus Design

For the psychophysics, we kept the frequency spectrum and stimulus level of the maskers the same as for the COR recordings, and presented them continuously throughout each threshold estimation. The probe level was then adjusted in order to derive probe detection thresholds for the different masker types (1/8th-octave, 1-octave and notched-noise masker). Detection thresholds with the notched-noise masker were also measured for both cats and humans.

### Methods: Cat Psychophysics

Four male cats were trained and yielded psychophysical data; they were neutered to reduce aggressive behaviour and thereby enable group housing. Cats 1–3 yielded complete data in all conditions. Cat 4 was tested with all the masker conditions but only with the 8-kHz probe tone. The cats were 5–16 months of age at the beginning of training. Weights ranged from 4.1 to 5.9 kg during most of the data collection. Food was restricted on psychophysical training days (5 days/week). On those days, cats received all or most of their food as moist food provided as reward during the psychophysical task. They generally worked until sated, but dry food and water was offered freely for ~ 1 h after each testing session. On weekends, cats received free access to dry food for 3 h per day. Water was freely available in the housing area.

The cat psychophysical experiments were conducted in a double-wall sound-attenuating chamber, having interior dimensions of 2.6 × 2.6 × 2.5 m, and lined with SONEXone adsorbent foam to suppress sound reflections. The cat sat or stood on an elevated platform in the centre of the chamber. A harness restrained the cat to the platform while permitting free movement of the head and limbs. Cats generally maintained an orientation of the head and pinnae toward the sound source, located in the front of the chamber. A pedal was positioned in front of the cat for psychophysical responses, and a feeder was present that could deliver small portions of canned commercial cat food. Acoustic stimuli were presented through a 3″ co-axial loudspeaker (Fostex FF85WK) in a bass reflex enclosure located 1.2 m in front of the cat. Similar to the COR recordings, stimulus generation, data acquisition, and experimental control used TDT equipment controlled by custom MATLAB scripts on a Windows-based personal computer. The speaker was calibrated daily in the same way as for the COR recordings. The detection task used a hold-release design that was derived from that described by May and colleagues (1995) and adapted in our previous study (Javier et al. 2016). Each trial began by the operator illuminating a green light near the location of the sound source. The green light signalled to the cat that it could press and hold a pedal to activate a continuous sound consisting of just the pink noise (on no-mask trials) or the pink noise plus the 1/8th- or 1-oct masker. After a delay varying randomly among values of 2, 3, 4 and 5 s, the probe sound was initiated, lasting to the end of the trial; the various probe-onset conditions are referred to, respectively, as Hold 2, Hold 3, Hold 4 and Hold 5. A release of the pedal earlier than 1000 ms prior to probe onset was recorded as an “early release” and was not scored. A release 1000 to 0 ms prior to probe onset was scored as a “false alarm”. A release of the pedal within 0 to 1000 ms after probe onset was scored as a “hit”, and the cat received a reward consisting of a small portion of food. Releases later than 1000 ms after probe onset were scored as “misses”. Early releases, false alarms, and misses were punished by a 2-s time-out period signalled by a flashing blue light in which no trial could be initiated. The masker was terminated after each pedal release, or 1000 ms after the end of the hit window, to be restarted by the next pedal press.

**Fig. 6:**
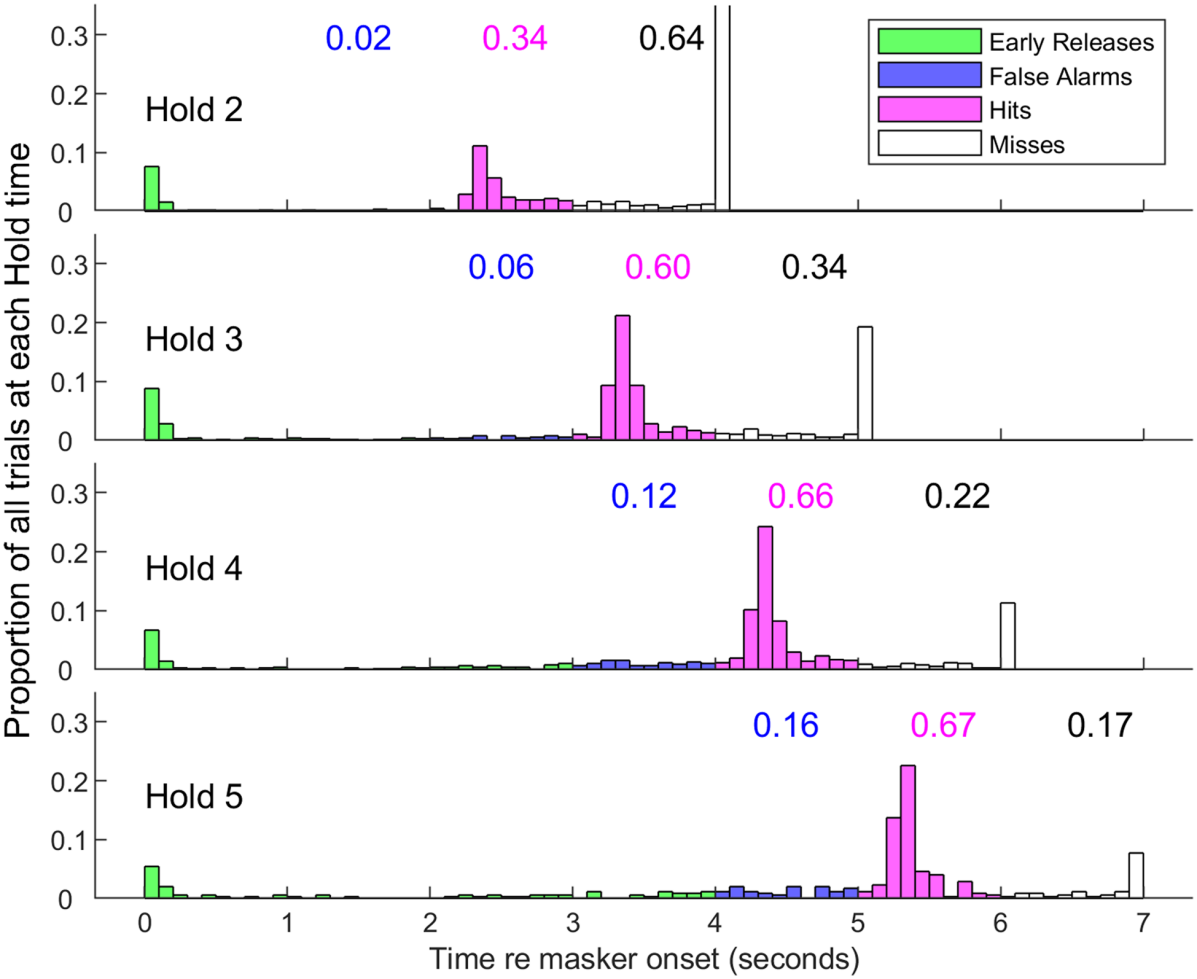
Response latencies for Cat 2. Data are combined across all tested masker conditions, probe frequencies and probe levels. Hold time was the time in seconds from masker onset to probe onset. Hold times of 2–5 s are represented in the 4 panels. Green, blue, magenta and white bars represent the proportions of trials that were scored as early release, false alarm, hit and miss, as described in the text. Latencies longer than 1 s after the end of the hit window are combined in one bar for each hold time. Early releases were not included in measures of percent correct. Blue, magenta and black numbers indicate the respective proportions of scored trials (i.e. not early releases) for each hold number

Figure 6 shows the latencies of pedal-release times for one cat in the various Hold conditions; data here are combined across all masker conditions and probe frequencies and levels, and colours indicate how the various latencies were scored. The histograms in the figure include trials both in which the probe level was above threshold and in which the probe level was below threshold, which accounts in part for the large number of misses. Note that the false-alarm response-time window for Hold N + 1 trials coincided exactly with the hit window of Hold N trials. For that reason, we used the false alarms on Hold N + 1 trials as catch trials (i.e. no probe) for the Hold N trials. Hold 5 trials were used only as catch trials for Hold 4; hits on Hold 5 trials were rewarded but were not included in the computation of hits. Hit rates were counted separately for each masker type, probe frequency, and probe level condition, whereas false alarm rates were counted separately for each masker type but combined across all probe frequencies and levels. The rationale for combining false alarms across probe conditions is that the cat produced a false alarm or correct rejection prior to the onset of the probe, rendering any probe properties irrelevant for the count of false alarms. The proportions of false alarms and hits relative to misses tended to increase with increasing hold time, which we attribute to cats’ impatience to receive the food reward. We aimed to mitigate any bias that might have been introduced by those trends. For that purpose, we computed hit and false-alarm proportions rates separately for each hold time; those numerical values are given in the illustration. For each block of trials, a false-alarm rate was computed separately for Holds 3, 4 and 5, those rates were weighted according to distribution of Holds 2, 3 and 4 tested for each probe condition, and then a weighted average false alarm rate was computed.

The sound levels of the 1/8-oct and 1-oct maskers were 65 dB SPL and 73 dB SPL, respectively. Those levels were chosen to equalise the energy within the human-sized auditory filter; the human equivalent rectangular bandwidth (ERB) centred on 8 kHz was estimated to be 888 Hz (Glasberg and Moore 1990). In the present psychophysical experiments, probe levels on each trial were controlled by the operator and were adjusted adaptively to fill out psychometric functions spanning sub-to-suprathreshold levels in 2-dB increments. Test sessions were carried on across days until enough trials were completed at each masker, probe, and level condition, with a minimum of 2 probe levels tested above and 2 below the final threshold. A minimum of 15 trials was obtained at probe levels that were well below or above the eventual measured threshold and a larger number was obtained at levels within 2 dB of that threshold. Typically, the probe frequency and masker condition were held constant throughout a session, with only the probe level varying among trials.

Performance was given by the unbiased maximum proportion correct, *P(c)max* (Green and Swets 1966; Macmillan and Creelman 1991). For each stimulus condition:

$$d^\prime=z\left(P_{hit}\right)-\left(P_{false \;alarm}\right),\;\mathrm{and}$$

$$P\left(c\right) max=\Phi \left(d^\prime/2\right),$$

where *P*_*hit*_ and *P*_*false alarm*_ are the proportions of hits and false alarms, *z* is the transform to standard deviates, *d’* is the unbiased sensitivity index, and *Φ* is the normal distribution function. Logistic equations were fit to plots of *P(c)max* versus signal level, and the thresholds for detection of tones were given by the interpolated signal level at which the logistic curve crossed *P(c)max* = 0.69; that value corresponds to *d’* = 1. We estimated the chance level for *P(c)max* by performing a permutation test in which we randomised the association between Hold times and pedal releases. That test showed the 95th percentile of *P(c)*_*max*_ given chance performance as 0.61.

### Methods: Human Psychophysics

Group B of the human participants (minus author AJH, N = 11) undertook the psychophysical procedures and completed all the psychophysical trials. Experiments were conducted with the listener seated in a sound-attenuating booth. In a similar manner to the COR recordings, all stimuli were generated using custom MATLAB scripts, played through an RME Fireface UCX sound card, TDT headphone drivers, and a monaural ER2 insert phone. The particular ear that was tested was balanced among listeners (6 left, 5 right). Stimuli were calibrated using an ear coupler (GRASS) and a Hewlett Packard 303 HP3561A dynamic signal analyser.

Masker levels were held at 60 dB SPL for the 1/8th-oct masker and 67.9 dB SPL for the 1-oct and notched-noise masker; those masker levels were equal to those used in the COR recordings. A 2-alternative forced choice adaptive procedure was used in which the probe level was increased after every incorrect response and decreased after every two consecutive correct responses. The change from increasing to decreasing probe level or vice versa was defined as a turnpoint. Each run of the procedure finished after 8 turnpoints, with the threshold for that run being computed from the mean of the last 6 turnpoints. The step size was 6 dB for the first 2 turnpoints, and 2 dB thereafter. The 2- down 1-up adaptive procedure estimates the signal level yielding 70.7 % correct (Levitt 1971). A noise stimulus consisting of the pink noise with or without the 1/8th-oct, 1-oct or notched-noise masker was presented continuously throughout each adaptive track. The probes were 50-ms long with 2-ms sine-squared ramps, as in the COR recordings. In each trial, two windows flashed successively on a computer screen, and the probe was presented randomly during one or the other window. The listener’s task was to report the time window that coincided with the probe. Feedback was given to the listener after each trial. Thresholds were computed from the average of 2 runs, or 3 if there was more than a 5-dB difference between the first two thresholds.

### Results

#### Bandpass Maskers

**Fig. 7:**
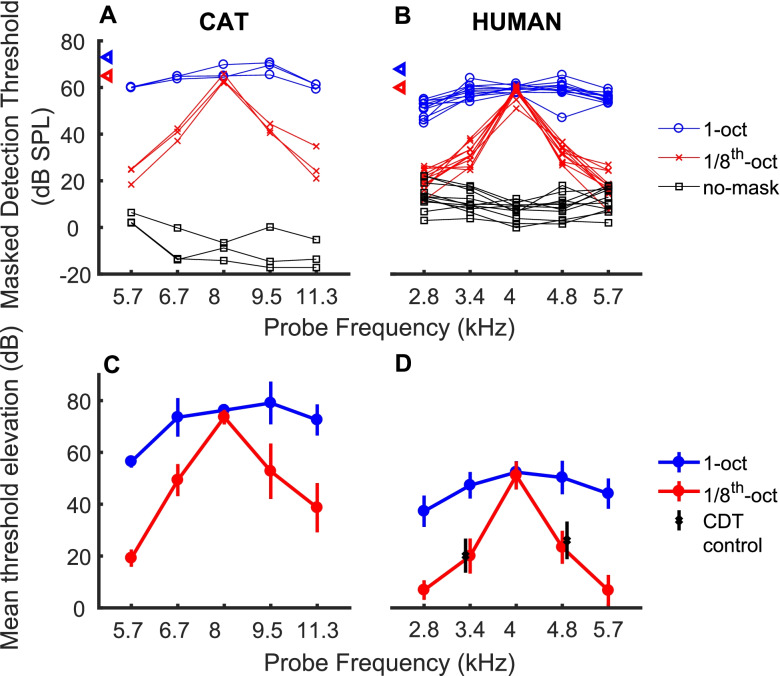
**A**, **B** Masked and unmasked detection thresholds. Each line represents thresholds of one cat (**A**) or human (**B**) listener. Blue, red and black lines denote 1-oct, 1/8-oct, and no masker conditions, respectively. Blue and red triangles on the ordinate indicate RMS masker levels. C-D. Mean threshold elevations. Mean values represent the mean of the masked threshold for each condition and listener minus the no-masker threshold for that listener. Symbols and error bars indicate means and standard deviations. Blue and red indicate 1- and 1/8-oct masker conditions, respectively. Black symbols and error bars near probe frequencies of 3.4 and 4.8 kHz in **D** indicate a condition in which an extra 1/8th-oct masker was added to mask cubic distortion products, as discussed in the text. Those symbols are displaced slightly horizontally to improve visibility

Detection thresholds in the 1/8-oct and 1-oct masking conditions are shown for the 3 individual cats that completed all the psychophysical conditions in Fig. 7A and for the 11 individual human listeners in Fig. 7B. For both species, thresholds were roughly constant across the ranges of probe frequencies in no-masker (black lines) and 1-oct-masker (blue lines) conditions. In contrast, masked thresholds dropped steeply at probe frequencies away from the centre frequency of the 1/8-oct masker (red lines). The unconnected triangles on the left axis of each panel show root-mean-squared (RMS) levels of the two maskers used for each species.

The masked thresholds for probe frequencies in the centre of the 1/8-oct and 1-oct maskers were broadly similar between cat and human listeners. The masked thresholds for cats were however ~ 5–10 dB higher overall than those for humans, attributable largely to the 5-dB difference in the masker levels that were used. The nonmasked thresholds for the cats were substantially lower than those for the humans: generally ≤ 0 dB SPL for the cats (Fig. 7A, black lines) compared to around + 10 dB SPL for the humans (Fig. 7B, black lines). We attribute the lower unmasked thresholds for the cats to the use of sound-field stimulus presentation for the cats compared to the use of unilateral insert earphones in the humans. In the open sound field, the cats benefitted from the acoustic gain provided by their pinnae, which is about 10–20 dB at 8 kHz for frontal sounds with the pinnae in a forward position (Wiener et al. 1966; Young et al. 1996; Tollin and Koka 2009). Also, the cats would have experienced some binaural summation (Hirsh 1948), which was not available to the human listeners, who listened monaurally. The unmasked thresholds at, for instance, 8 kHz (− 14.2, − 8.8 and − 6.6 dB SPL) agree well with previous reports of cat behavioural thresholds at that frequency (Elliot et al. 1960; Gerken and Sandlin 1977; Heffner and Heffner 1985).

Mean data for the cats, expressed as the elevations of thresholds above the unmasked condition for each cat, are shown in Fig. 7C. Both the 1/8-oct and 1-oct maskers produced > 70 dB elevation of the threshold of the 8-kHz probe. The threshold elevation by the 1-oct masker was roughly constant across probe frequencies within the masker passband. A one-way ANOVA of the threshold elevations in the 1-oct masker showed a significant effect of probe frequency (*F*(4,10) = 6.9, *p* = 0.0064), but a post hoc test showed that only the value at 5.7 kHz differed significantly (*p* < 0.05) from the values at the other individual probe frequencies. In contrast, threshold elevation by the 1/8-oct masker decreased markedly as the probe tones were shifted above or below the 8-kHz centre frequency. A one-way ANOVA of the 1/8-oct threshold elevations showed a highly significant effect of probe frequency (ANOVA, *F*(4,10) = 22.7, *p* < 0.001), and all of the values at off-centre frequencies were significantly lower than that at 8 kHz (post hoc comparison of each off-centre frequency compared to 8 kHz, *p* < 0.05). Importantly, a two-way ANOVA revealed a significant frequency-by-masker interaction (*F*(4,20) = 6.3, *p* = 0.002). Even the probe frequencies closest to the masker centre frequency (i.e. 6.7 and 9.5 kHz) showed significantly less masking by the 1/8-oct masker than by the 1-oct masker (2-way ANOVA of those 2 frequencies and 2 masker conditions: *F*(1,8) = 27.8, *p* < 0.001 for masker condition; *F*(1,8) = 0.9, *p* = 0.38 for frequency). The dependence of masked psychophysical thresholds on probe frequency clearly distinguished between the two masker bandwidths.

The mean threshold elevations for the humans (Fig. 7D) varied with probe frequency and masker type in a way that was broadly similar to that of the cats. As was observed in the cats, threshold elevations were relatively constant across the passband of the 1-oct masker (blue line) except for the value at the lowest probe frequency (2.8 kHz), whereas threshold elevation by the 1/8-oct masker dropped away sharply at off-centre probe frequencies. For probe frequencies most remote from the 1/8 oct. masker thresholds were close to, and may have been limited by, detection thresholds in quiet. A two-way ANOVA showed main effects of probe frequency (*F*(4, 100) = 92, *p* < 0.001) and of masker bandwidth (*F*(1,100) = 519, *p* < 0.001) and, importantly, a significant interaction between probe frequency and condition (*F*(4, 100) = 32, *p* < 0.001). Masking by the 1/8-oct masker was significantly less than that for the 1-oct masker even for the off-centre probe frequencies nearest the 4-kHz centre frequency (2-way ANOVA, *F*(1,40) = 210, *p* < 0.001 for masker condition; *F*(1,40) = 2.9, *p* = 0.098 for frequency). We considered the possibility that cubic distortion products formed between the 1/8-oct masker and the 3.36 or 4.76 kHz probes might have influenced the thresholds masked by the 1/8-oct masker. Adding a narrowband masker centred on either of those frequencies (1/8th-octave wide, 20 dB below the 4-kHz 1/8th-oct masker), however, had only a negligible effect on masked thresholds for probes at those frequencies in the 1/8-oct masker condition (Fig. 7B, black X’s with error bars).

The cat and human mean threshold elevations shown in Fig. 7 differed from each other in that the cats showed substantially greater masking, by more than about 20 dB at the centre frequency, than did humans. Also, in the 1/8-oct masking condition with probe frequencies ½-oct on either side of the centre frequency, the cat thresholds were ~ 20–40 dB above the unmasked thresholds whereas the human thresholds dropped to within 10 dB of the unmasked thresholds. There was only a small difference in the masker levels between the cats and humans (5 dB SPL for the 1/8th octave noise), so the large difference in overall masking is likely due to the additional gain produced by the cats’ pinnae in free-field presentation; as noted above, this would have reduced thresholds in quiet but, because it would affect the maskers and probes equally, would have had little or no effect on masked thresholds.

#### Notched-Noise Maskers

**Fig. 8:**
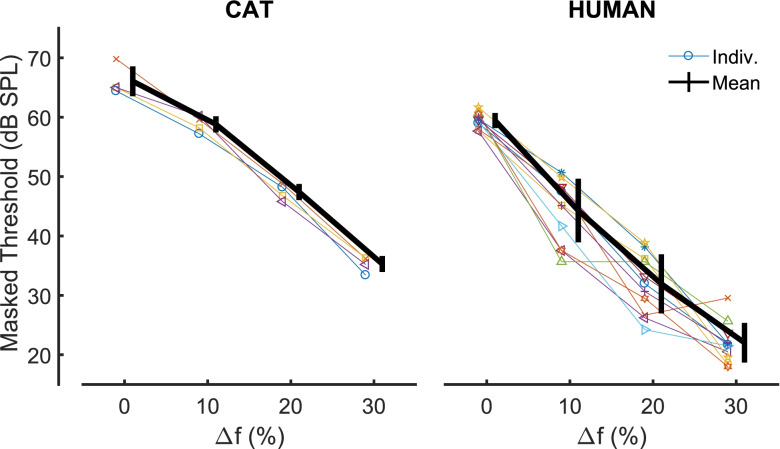
Masked thresholds in notched-noise conditions. Left panel represents individual data (thin lines) and mean (thick line) from 4 individual cats. Right panel shows data collected in human participant group B. Individual colours/symbols match those of Figs. 3 and 5

In cats, we measured thresholds for detection of 8-kHz probe tones in 1-oct-wide noise bands having notches of Δ*f* = 0, 10, 20, and 30 %, as described in the Methods. In humans, thresholds for 4-kHz probes were measured similarly for notches centred on 4 kHz for the purpose of validating results relative to published reports and to compare them to COR results obtained using similar methods and stimuli. The thresholds for the 4 cats are plotted in Fig. 8 along with those for the 11 human listeners; for each species individual data are shown by coloured lines and symbols with the mean plotted as a thick black line. Again, masked thresholds for cats were higher than for humans: for the 0 % notch width the average difference was 6.6 dB, 5 dB of which could be accounted for by the difference in masker sound levels. Thresholds decreased more gradually with increasing notch width for cats than for humans, so that at the largest notch width, the average difference in masked thresholds between species was 13.2 dB. A 2-way univariate ANOVA revealed significant main effects of species (reflecting the higher overall thresholds for the cat: *F*(1,52) = 190.4, *p* < 0.001) and of notch width (*F*(3,52) = 136.0, *p* < 0.001). The notch width X species interaction was also significant (*F*(3, 52) = 3.5, *p* = 0.021), reflecting the shallower function relating threshold to notch width for cats compared to humans.

The following single-parameter symmetrical roex(p) filter function was fit to the mean data from feline or human listeners:

$$W\left(g\right)=\left(1+pg\right) {e}^{-pg},$$

where *W* is the filter weight, *g* is the normalised distance from the center frequency, and *p* is a fitted parameter that controls the steepness of the filter (Patterson et al. 1982). Those fits yielded equivalent rectangular bandwidths (ERBs) of 477 ± 16 Hz (mean and s.d. of 10,000 bootstrap samples) for the 11 humans at 4 kHz and 1087 ± 34 Hz for the 4 cats at 8 kHz. The human value is close to the value of 456 Hz estimated by the equation proposed by Glasberg and Moore for an ERB centred on 4 kHz (Glasberg and Moore 1990; their Fig. 3). That equation gives an estimated human ERB centred on 8 kHz of 888 Hz. Hence, the ERB for the cat was greater than obtained previously for human listeners. Given a 1-oct masker band centred on 8 kHz, one would expect 0.9 dB more power to fall within our estimated feline ERB of 1087 Hz than within the 888-Hz human ERB at that centre frequency (i.e. 10log_10_(1087/888)). Note that in both species we estimated auditory filter bandwidths using symmetrical notches, and that the measurements could largely reflect the low-frequency slope of the filter, which is typically broader than the high-frequency slope (e.g. Irino and Patterson 1997; Baker and Rosen 2006).

## Discussion

### Comparison with Previous Scalp-Recorded Measures of Tonotopic Selectivity

We are aware of three types of method that have been used to measure selectivity with scalp potentials, all of which were developed in experiments with human participants. One paradigm measures the response to a tone of a given frequency in the presence and absence of intervening tones at varying frequency separations (Butler 1968, 1972; Picton et al. 1978; Näätänen et al. 1988; de Boer and Krumbholz 2018). This has led to mixed results. Some authors reported very broad tuning (Butler 1968, 1972; Picton et al. 1978). For example, Butler (1972) found that the COR to 250-Hz tones presented every 5 s could be reduced by intervening 8-kHz tones. One feature of this method is that there is a (usually long) silent gap between the test and intervening tones, so that there is no masking per se between the intervening tones and test tones at the level of the cochlea or auditory nerve. Rather, the method relies on adaptation at multiple, more-central, stages of the auditory system, including at the cortex. Differences in stimulus parameters may cause the onset response to be dominated by different classes of neurons, which may in turn differ in their tonotopic selectivity. Indeed, the study by Näätänen et al. (1988) used a much shorter SOA of 500 ms and reported considerably sharper tuning than studies that used longer SOAs (e.g. Butler 1972). The measures used here involve masking of the neural response to the probe at the level of the cochlea and auditory nerve, and used a fairly short SOA of 1 s.

A second method is to change the frequency of a tone part-way through a stimulus, and to measure the size of the resulting COR as a function of the frequency separation between the two tones. He et al. (2012) measured this “acoustic change complex (ACC)” in 12 normal-hearing adults in response to changes in the frequency of a 500-Hz tone. The mean threshold frequency change was 5.8 Hz, equal to 1.4 % of the baseline frequency. Although larger than the psychophysical thresholds obtained in the same subjects, this value is much smaller than the smallest frequency separation of 19 % (i.e. 1/4th oct) between the probe and the centre frequency of the 1/8th-oct masker in our experiments. The ACC is the only scalp-recorded measure of cortical selectivity that we are aware of that has also been applied to a nonhuman species: Presacco and Middlebrooks (2018) obtained pure-tone ACC thresholds of between about 1 and 5 % in the sedated cat and showed that these thresholds were similar to psychophysical measures reported previously for the same species. The ACC can also be elicited in CI users by changing the stimulating electrode mid-way through a stimulus (e.g. He et al. 2014; Mathew et al. 2016). An advantage of the ACC measure over our masking method is that, because only one stimulus is present at any one time, it avoids complications associated with masker-probe interactions such as beating and cochlear distortion products for acoustic stimulation (Moore et al. 1984) and charge interactions for electrical stimulation (Cosentino et al. 2015). A disadvantage is that an ACC can be generated to changes in intensity and/or loudness, making it important to loudness-balance the stimuli. Loudness levels can vary substantially from electrode to electrode in CIs, especially for focussed modes of stimulation (Bierer and Faulkner 2010), making the procedure time-consuming in humans and hard to achieve in animal experiments. As we wished to generalise our method to CI experiments with focussed stimuli, this was one reason for not choosing the ACC paradigm.

Third, some authors have measured electroencephalography (EEG) or magnetoencephalography (MEG) responses to the onset of tones presented in noise containing different notch widths, analogous to our COR measures with notched-noise maskers (Sams and Salmelin 1994; Kauramäki et al. 2007; Okamoto et al. 2007). Those studies were primarily interested in the effects of attention, but included one condition similar to ours in which the subjects listened passively. Kauramäki et al. (2007) used EEG and measured the global field power of the N100 response to a 1-kHz tone presented in quiet or in noise containing notches of different widths centred on 1 kHz. Their COR increased with increases in Δf up to 25 % at which point it was similar to that in the no-mask condition. This differs from our notched-noise experiment in which a substantial (6 dB) reduction in COR was observed even at the Δf of 30 %. In addition to using lower noise and probe levels than here, Kauramäki et al. increased notch width by progressively removing portions of the masking-noise spectrum, such that its total bandwidth and overall power decreased with increasing notch width. The same was true of the study by Sams and Salmelin (1994), who measured the MEG N100m response to the onset of 1- and 2-kHz tones in notched-noise backgrounds. They observed quite variable and sometimes nonmonotonic functions relating the N100m to notch width. Our protocol differed from that of the preceding two studies in that we maintained a constant width of the upper and lower masker bands as they were separated to produce progressively wider notches, consistent with the stimuli used in psychophysical notch-noise-masking experiments described here and elsewhere. Okamato et al. (2007) found that the N1m to amplitude-modulated 1-kHz tones masked by notched noise increased up to the maximum notch width studied, but this corresponded to only an 8 % distance between notch edge and probe frequency, so we do not know how the N1m would have varied over wider ranges of notch widths.

### Cochlear Distortion Products

A stimulus consisting of two primary frequency components f1 and f2 can produce cochlear distortion products (DPs), the largest of which are typically the quadratic and cubic DPs having frequencies of f2-f1 and 2f1-f2, respectively, and that can sometimes be perceived by listeners (Goldstein 1967; Hall 1972; Smoorenburg 1972a; Zwicker 1979, 1981). Although they are usually studied using pure tones, DPs can also arise from the combination of a pure tone and a narrow band of noise, such as the 1/8th-octave maskers used here. Our pink noise encompassed the predicted frequencies of the quadratic DP for all masker and probe combinations, but, for the 1/8th-octave masker this was true for the cubic DP (CDP) only when the signal frequency was 0.5 octaves below the masker centre frequency.

The amplitude of the CDP is generally largest with small f2/f1 ratios and (for human listeners) becomes inaudible at ratios of between about 1.4 and 1.6 (Smoorenburg 1972b). We therefore think it unlikely that the CDP would have contributed to the COR observed for masker-probe separations of plus or minus 0.5 octaves, corresponding to a ratio of 1.41; furthermore, when the probe was 0.5 octaves above the signal the CDP would have been masked by the pink noise. However, the ± ¼-octave separation used for two probe frequencies corresponds to a ratio of 1.18 at which the CDP can be large. For our human participants, the centre frequency of the CDP for 3364-Hz probe would have been 2728 Hz, which is more distant than the 3364-Hz signal from the 4000-Hz masker, and so could have in principle contributed to the COR. We conclude that the CDP may have contributed to the COR for signals that were ¼ octave below the masker centre frequency but was unlikely to have done so for other signal frequencies. In the cat, we performed a control experiment with one additional cat with the pink noise cutoff extended to 7190 Hz (i.e. above the expected CDPs at 5454 and 6486 Hz, because of the doubling of the frequencies in the cat). That noise reduced the response to the 8000-Hz probe in the absence of any other masker, so we reduced its level by 10 dB, added it to the 1/8th-oct masker, and measured the COR to each masker/frequency combination. This showed a pattern of COR with probe frequency that was typical of those observed in the main experiment.

### Excess Masking

The psychophysical masking experiments with cats found that the 1/8-oct band at 65 dB SPL produced about the same amount of masking at 8 kHz as did the 1-oct noise band, which had a level of 70 dB SPL for five cats and 68 dB SPL for two cats. When we converted these thresholds to signal-to-masker ratios (SMRs) at the output of the cat auditory filter, using the estimates of filter ERB obtained from the notched-noise measurements, we found that the SMR was on average 1.9 dB higher for the 1-oct than for the 1/8-oct masker. This “excess masking” was statistically significant (*t*(3) = 3.96, *p* = 0.029). A similar analysis for the human listeners (and using the human ERB) found no significant difference. However, a two-way ANOVA on the masked thresholds for the probes at the masker centre frequency, relative to the masker level per species-specific ERB did not find a significant effect of species or a species X masker interaction. Hence, although we found significant excess masking only for the cat, the absence of a significant interaction means that we have no evidence that the excess masking differed significantly between species*.*

### Comparison of Psychophysical and COR Measures of Selectivity

#### Narrowband vs Wideband Maskers

**Fig. 9:**
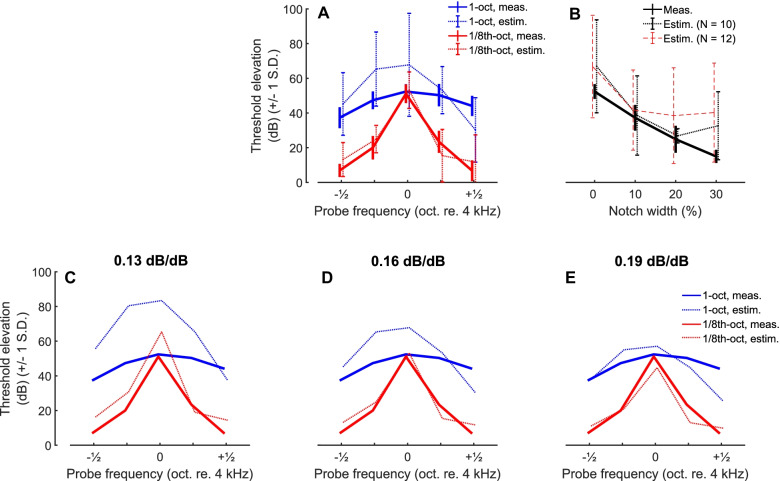
Comparison of psychophysical and COR results in humans. **A** Noise-band experiment. Thick lines show psychophysical results, and dotted lines the COR estimate obtained by dividing the COR masking (in dB re unmasked, group A) by 0.16, the average slope of the unmasked probe level growth measured in group B. **B** Notched-noise experiment. Solid line shows the psychophysical results, red dashed line shows the COR estimate averaged across all participants (*N* = 12). Black dashed line shows the results with the two outlier participants removed (*N* = 10). **C**–**E** Same analysis as **A** (without the error bars for visibility) when using different slopes: 0.13 dB/dB (**C**, lower 5 % confidence interval value), 0.16 dB/dB (**D**, average slope) and 0.19 dB/dB (**E**, upper 95 % confidence interval value)

To compare the pattern of results obtained with masked thresholds and with COR differences, it is important to use the same dependent variable in both cases. Figure 9A compares the COR (participant group A) and psychophysical measures of tonotopic selectivity (group B) by plotting the psychophysical threshold elevation together with estimates of that threshold elevation derived from the COR measures, for probes presented against 1/8th-oct. (red) and 1-oct. (blue) maskers. The COR estimate for each subject was calculated by dividing that subject’s COR masking (in dB re unmasked) from experiment 1a by 0.16, which was the average slope of the level-growth function (Fig. 3). By doing so, we converted the reduction in the COR produced by each masker to the reduction in stimulus level (in quiet) that would produce an equivalent reduction in COR. This places the COR measures on the same stimulus dimension as the psychophysical thresholds, namely stimulus level in dB SPL, thereby allowing a more direct comparison between the two measures. An even more direct comparison might have been to measure amplitude growth functions in the presence of each masker so as to estimate the increase in probe level needed to restore the COR to that produced by the original probe level in quiet, but unfortunately that information is not available and may have required the presentation of uncomfortably loud probes.

Using the method described above, the predicted and obtained psychophysical thresholds agree very well for the 1/8th-oct masker (red, Fig. 9A). This is somewhat less the case for the 1-oct masker (blue, Fig. 9A), where the COR estimates tend to reduce with increasing distance from the centre frequency (4 kHz) whereas the psychophysical function is much flatter. As a result, the COR measure generally overestimates the amount of psychophysical masking, except for the highest probe frequency where it underestimates it. These trends were confirmed statistically as follows. We first performed an ANOVA with masker bandwidth and probe frequency as repeated measures and “estimated vs obtained” thresholds as a between-subjects factor. As expected there were highly significant main effects of probe frequency and of masker bandwidth and a highly significant interaction between these two factors (probe frequency: *F*(4,76) = 56.2, *p* < 0.001; masker bandwidth: *F*(1,19) = 183.2, *p* < 0.001; interaction *F*(4, 76) = 13.7, *p* < 0.001). More importantly, there was a significant three-way interaction between the factors “estimated vs obtained”, probe frequency, and masker bandwidth (*F*(4, 76) = 5.6, *p* < 0.001), reflecting the fact that the variation in the COR-estimated thresholds with probe frequency closely followed that observed for the psychophysical thresholds for the 1/8th-oct masker, but less so for the 1-oct masker. Note that the exact values of our predictions will depend on the estimate of the slope of COR level-growth function, which differed somewhat across listeners. Figure 9C, D and E show the predictions obtained with slopes of 0.13, 0.16 and 0.19 dB/dB, corresponding respectively to the lower 5 % confidence limit, average, and upper 95 % confidence limits of the slopes obtained from our participants. This affects both the size of the predicted threshold variation and the extent of its variation with frequency, but the overall pattern, including the significant interactions between masker type, probe frequency, and “predicted vs obtained” remains (slope = 0.13, *F*(4,76) = 4.5, *p* = 0.004; slope = 0.19, *F*(4,76) = 6.9, *p* < 0.001).

**Fig. 10:**
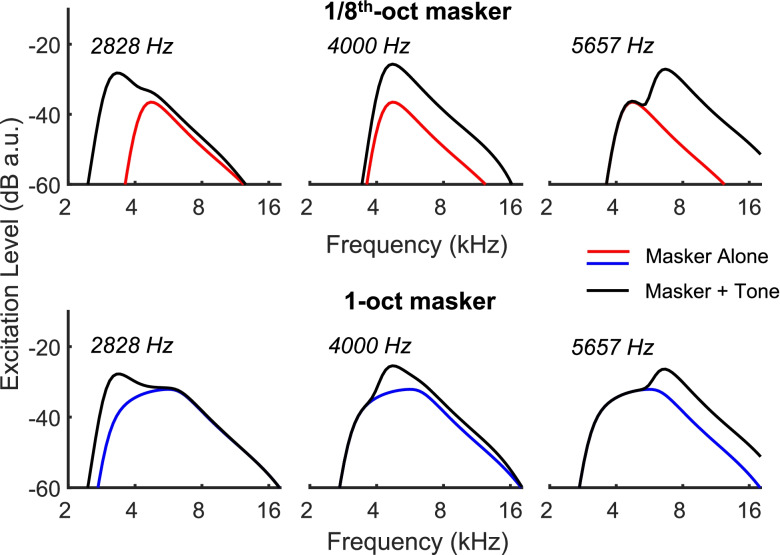
Simulated excitation patterns for masker-alone (blue/red lines) and masker plus probe (black lines). Top and bottom rows are for the narrow-band and wideband-masker conditions respectively. Probe frequency was 2828, 4000 and 5657 Hz in the left, middle and right plots, respectively. All simulations were generated using the nonlinear gammachirp auditory filter bank implemented in the AIM-MAT software package (Gaudrain et al. 2015)

The highly significant interaction between “obtained vs predicted” and probe frequency for the 1-oct masker reflects the fact that whereas psychophysical masking was broadly similar at all frequencies, the 1-oct masker was less effective at reducing the COR to probes near the edges (and especially at the upper edge) of the 1-oct masker (dotted blue line in Fig. 10A). Some insight into this finding comes from simulations of the excitation patterns produced by adding different-frequency probes to the 1-oct masker at the + 10 dB SNR used in the COR experiments and illustrated in the bottom row of Fig. 10. These simulations used the nonlinear gammachirp filterbank (Irino et al. 2006; Gaudrain et al. 2015) implemented in the AIM-MAT software package (Gaudrain et al. 2015). Because basilar-membrane nonlinearity results in a marked upward spread of excitation, there is a wider range of auditory-filter frequencies that convey a greater-than-masker probe response (black line versus blue line) to the 5657-Hz tone than with the 2828- and 4000-Hz tones. This arises because the probe has a higher level (per ERB) than the noise and because the auditory filter bank is nonlinear; it would occur to a much lesser extent in the psychophysical experiments, where the SNR at threshold was less than 0 dB (Fig. 8).

Figure 10 also suggests that the COR should predict more masking of the 4-kHz probe by the 1-oct (bottom row) than by the 1/8th-oct masker (top row). Figures 9A and 10D show that this was true numerically for human listeners, but the difference was not statistically significant (*t*(9) = 1.63, *p* = 0.135). Significant excess masking of 3–5 dB was however observed in the cat COR measurements.

To summarise, it may well be that, in both species, the positive probe-to-masker ratio used for the COR experiments reduces masking of the COR for tones on the upper edge of a 1-oct masker and might also account for the excess COR masking observed in the cat, for 8-kHz tones masked by 1-oct vs 1/8th-oct maskers. These suggested effects can be at least qualitatively explained using a nonlinear auditory filterbank. The variability in level-growth slope estimates (0.11 to 0.70) in the cats, combined with the smaller number of cats tested, did not permit reliable estimates such as achieved for humans in Fig. 9A. This precluded quantitative comparisons between COR and psychophysical results in cats. In addition, we note that psychophysical experiments with humans have shown that, for narrowband maskers, listeners can detect a tonal signal by virtue of the resulting change in the shape of the masker envelope, and that there is some evidence that the use of this cue is greater for narrow than for broad masker bandwidths (Richards 1992; Richards and Nekrich 1993). This could in principle lead to masking by a narrowband noise being slightly lower than that produced be a wider-band noise having the same level in an ERB centred on the probe frequency. It is not known, however, whether this envelope-shape cue can affect the COR, and comparable psychophysical data are not available for the cat.

#### Notched-Noise Maskers

Figure 9B compares the notched-noise COR measurements made with humans with the psychophysical thresholds obtained with the same listeners. Psychophysical threshold shifts relative to the no-mask condition were predicted from the COR data by dividing the reduction of the COR amplitude, in dB, by the slope of the COR input–output function (in dB/dB) for unmasked probes. The considerable across-participant variability in both the masked CORs and the input–output function for unmasked tones warrants some caution in interpreting these results. Nevertheless, it can be seen that the COR data do a fairly good job of predicting the psychophysical masking for Δf of 0 %, 10 % and, when the two outliers are removed, 20 %, but that the amount of predicted masking does not decrease at the 30 % notch width, unlike the psychophysical data

### Across-Species Differences in Tonotopic Selectivity

Our observation that the feline auditory filter is approximately 22 % wider than that of humans is consistent with several previous reports. Using a band-widening procedure with cats, Pickles (1975) and Nienhuys and Clark (1979) estimated critical bands at 8 kHz as around 1520 or 1290 Hz, respectively. Those values are in line with our value of 1087 Hz, considering that critical-band estimates tend to be broader than ERBs obtained with notched noises. In another member of the Carnivore order, the ferret, a notched-noise study yielded an even wider ERB at 8 kHz of 1680 Hz (interpolated from values for 7 and 10 kHz reported by Alves-Pinto et al. 2016). We note that our study employed simultaneous masking, which tends to yield broader ERBs than do forward-masking procedures (Oxenham and Shera 2003; Sumner et al. 2018); however, simultaneous masking was also used in the other animal studies cited here (Pickles 1975; Nienhuys and Clark 1979; Alves-Pinto et al. 2016) as well as all the human studies considered in the derivation of an equation for ERB versus centre frequency by Glasberg and Moore (1990). Measurements of ERB using notched noise centred at 8 kHz in species less closely related to cats have yielded ERB values ranging from ~ 900 Hz (Evans et al. 1992), through 1377 Hz in macaque (Burton et al. 2018), to 1540 Hz in mouse (May et al. 2006).

## Summary and Future Applications

The COR method described here provides an objective measure of tonotopic selectivity that broadly agrees with psychophysical measures obtained in the same species (cats and humans) and with similar stimuli and methods. The COR presumably reflects all stages of auditory processing up to and including auditory cortex, thereby providing a measure that may be more tightly linked to perception than those obtained at more peripheral stages of processing such as masking patterns derived from electrically evoked compound action potentials (Biesheuvel et al. 2016; Cosentino et al. 2016; Garcia et al. 2021) in CI stimulation or from the adaptation of the Frequency Following Response in acoustic hearing (e.g. Gockel et al. 2015). The method can be applied without the extensive training needed to obtain reliable psychophysical thresholds from animals, making it of benefit to a wide range of applications including those evaluating novel stimulation methods (e.g. optogenetics, penetrating-nerve stimulation) and pharmaceutical interventions. It may also allow animals, e.g. those implanted with a CI, to be shared between multiple investigations in the same laboratory, without the concern that training an animal to perform one task might interfere with its performance on another. The link between perception and COR measurements may be improved by obtaining COR input–output functions for unmasked tones, which allows one to compare the two measures using the same dependent variable, namely stimulus level in dB.

Transferring the method to CIs and to novel (e.g. optogenetic) forms of stimulation should, we believe, be quite straightforward and indeed would avoid complicating factors such as the generation of cochlear distortion products and the nonlinearity of the auditory filter bank (Fig. 10). Additional technical issues would include, for CIs, the elimination of radiofrequency and electrical-pulse artefacts, but preliminary measurements from our laboratory have shown that this can be achieved. Although the difference between the shapes of the 1-oct and 1/8th-oct COR masking patterns was large, we do not know exactly how accurately the method would reflect smaller differences. However, differences in excitation-pattern width between different CI configurations can be substantial. For example, George et al. (2015) reported that excitation-pattern widths in the cat inferior colliculus were about 50 % wider for monopolar stimulation than for tripolar stimulation. We are therefore optimistic that the paradigm can be successfully applied to the study of tonotopic selectivity both in humans and in cats, and for varying forms of stimulation. The method is noninvasive and does not require extensive training, thereby making it applicable to longitudinal experiments and to those where testing time is limited.
